# Effects of supplementation of garlic with apple pomace or blackcurrant on the gastrointestinal microbial ecosystem of organic pigs after weaning

**DOI:** 10.1186/s12866-025-04247-2

**Published:** 2025-10-02

**Authors:** Kevin Jerez-Bogota, Martin Jensen, Ole Højberg, Nuria Canibe

**Affiliations:** 1 Department of Food Science, Aarhus University, DK-8200 Aarhus N, Denmark; 2 Department of Animal and Veterinary Sciences, Aarhus University, AU-Viborg, DK-8830 Tjele, Denmark

**Keywords:** Apple pomace, Blackcurrant, Garlic, Gastrointestinal, Microbiota, Microbial metabolites, Organic pigs, Postweaning

## Abstract

Weaning stress compromises piglet immune and digestive development, increasing susceptibility to *Escherichia coli* (ETEC) infections. We have previously shown that combinations of antibacterial plants alleviate postweaning diarrhea caused by ETEC. Here, we evaluated the effect of feeding garlic combined with either apple pomace or blackcurrant on the gastrointestinal microbial ecosystem of organic weaners three weeks after an ETEC challenge at weaning. The study involved two blocks of 32 pigs (7 weeks; 20 kg BW) per block housed in pairs. The pairs were randomly assigned to four treatments: non-challenged control (NC); ETEC-challenged control (PC); ETEC-challenged fed garlic and apple pomace (3% w/w each; GA); and ETEC-challenged fed garlic and blackcurrant (3% w/w each; GB). One pig per pen was euthanized on days 21–22 after weaning for digesta and mucosa sampling. Digesta was analyzed for bacterial counts (qPCR), microbial profiling (16S rRNA), and metabolites, while jejunal and ileal mucosa were assessed for antioxidant enzyme activity. The treatments did not influence the number of F18 fimbriae’s gene, *E. coli*, *Lactobacillus* and total bacteria in the gut digesta, nor the jejunal and ileal mucosa expression of OCNL, ZO-1, and *NF-κB* genes. Compared to NC, PC did not alter the overall gut microbiota composition but increased (*P* ≤ 0.05) *Campylobacter* and reduced *Roseburia *relative abundance. Compared to NC and PC, GA increased (*P* ≤ 0.05) *Weissella* relative abundance and reduced lactic acid and cadaverine concentrations in the stomach and jejunum. Both GA and GB increased (*P* ≤ 0.05) superoxide dismutase and glutathione peroxidase activities in the small intestinal mucosa. GA and GB increased (*P* ≤ 0.05) *Catenibacterium* and *Holdemanella* in the cecum and colon, with GB additionally increasing (*P* ≤ 0.05) short-chain fatty acid concentrations. Compared to NC and PC, GA increased (*P* ≤ 0.05) *Faecalibacterium* abundance in the cecum. GA and GB increased (*P* ≤ 0.05) the proportion of butyric acid in the colon. In conclusion, the combination of garlic with either apple pomace or blackcurrant increased the abundance of beneficial gut bacteria, leading to improved hindgut fermentation activity and oxidative status in the small intestine of pigs three weeks after weaning.

**Supplementary Information:**

The online version contains supplementary material available at 10.1186/s12866-025-04247-2.

## Background

The postweaning period in pigs is a critical developmental stage characterized by significant physiological and microbial shifts in the gastrointestinal tract (GIT). Following weaning, the gut microbiota undergoes substantial changes that can influence nutrient digestion, immune function, and overall health [1]. During this time, pigs are particularly susceptible to bacterial infections such as enterotoxigenic *Escherichia coli* (ETEC), leading to postweaning diarrhea (PWD). Managing PWD presents specific challenges in organic farming systems. Although pigs are weaned later in organic systems (typically at 6–7 weeks, compared to 3–4 weeks in conventional systems), they still experience health issues associated with the weaning transition [2]. Furthermore, organic regulations limit the use of common dietary strategies (e.g. feed additives, synthetic amino acids, antimicrobial drugs). One strategy to prevent bacterial infections and reduce the reliance on antibiotics is dietary supplementation with bioactive plant materials [3, 4].

Plant materials contain a variety of bioactive phytochemicals and dietary fiber, which can impact the host beyond their antibacterial effects through beneficial interactions with the gut microbiota and host tissues, leading to changes in microbial metabolite production and improved intestinal health. Such effects are particularly valuable in organic systems, where for example, limitations on feedstuff sources, synthetic amino acids and feed additives often lead to high dietary levels of crude protein (**CP**) that can negatively impact animal health [2]. Excessive CP intake can lead to increased protein fermentation in the gut, reducing nitrogen efficiency and contributing to oxidative stress in the intestinal mucosa, disrupting the GIT ecosystem [5, 6].

Garlic, renowned for its broad-spectrum antimicrobial effects due to allicin and other sulfur-containing compounds, also contains a substantial amount of fructans; prebiotic carbohydrates comprising approximately 75% of the garlic dry matter (DM) [7]. Garlic products have been explored for their potential to mitigate the effects of ETEC infections in pigs and their immunomodulatory properties [8–10]. Apple pomace, a byproduct of apple processing, is rich in dietary fiber and polyphenols and is known to promote beneficial gut bacteria and enhance gut barrier function in piglets [11, 12]. Similarly, blackcurrant is notable for its potent antioxidant effects and high content of organic acids and polyphenols [13]. Garlic, apple pomace, and blackcurrant, which are abundant in bioactive compounds such as organosulfurs, phenolics, and flavonoids, have demonstrated the potential to neutralize harmful substances, inhibit lipid peroxidation, and increase antioxidant enzyme production in the GIT [13–15]. Thus, on the basis of their individual compositions and properties, garlic, apple pomace, and blackcurrant can exert various effects on the gut microbial ecosystem. Combining these plant materials may lead to different interactions, including synergistic action, influencing their overall impact on gut health [16].

In an earlier report, we showed that dietary supplementation with garlic, combined with either apple pomace or blackcurrant, effectively alleviated PWD and modulated the fecal microbiota in ETEC-challenged piglets, particularly during the first week after weaning [8]. The adverse effects of the ETEC infection were most pronounced within the first week postweaning but substantially diminished by three weeks after weaning [8]. However, the extended effects of the antibacterial plant combinations on the overall GIT ecosystem, specifically after three weeks of supplementation in organic pigs previously challenged with ETEC, remain unexplored.

Thus, the aim of this study was to investigate the impact of dietary supplementation with garlic, in combination with either apple pomace or blackcurrant, on the gastrointestinal microbiota, microbial metabolites, and antioxidant capacity and barrier function of the small intestinal mucosa in organic pigs three weeks after ETEC challenge at weaning. We hypothesized that the three-week dietary intake of the antibacterial plant combinations would beneficially influence the GIT ecosystem by selectively promoting the growth of beneficial bacteria and thereby modulating fermentation processes that can prevent detrimental processes such as oxidative stress and intestinal barrier disruption.

## Materials and methods

The animal experiment was performed at the Department of Animal and Veterinary Sciences, Aarhus University, Denmark. Animal care, housing, and euthanasia were in accordance with Danish laws and regulations governing the humane care and use of animals in research. The experimental procedures were approved by the Danish Animal Experiments Inspectorate, Ministry of Food, Agriculture and Fisheries, Danish Veterinary and Food Administration (License 2017–15-0201–01270).

### Experimental animals, design, and treatments

The study consisted of two consecutive blocks conducted sequentially, each lasting three weeks. A total of 64 organic weaners (7 weeks old; BW: Mean = 20.1 kg, SD = 1.9 kg) born from eight organically raised sows were included in the study (32 pigs in each block; 8 piglets per sow). The sows (TN70 Topigs genetics; bred to Duroc) were multiparous and tested positive for homozygote carriers of the α-(1,2) fucosyltransferase dominant gene encoding ETEC F18 fimbriae receptors (VHL genetics, Wageningen, The Netherlands).

The pigs were obtained at weaning and housed in pairs of littermates within individual pens, totaling sixteen pens per block. Pens were randomly assigned to one of 4 treatments: non-challenged control fed a standard diet (negative control, NC); ETEC-challenged fed a standard diet (positive control, PC); ETEC-challenged fed a diet supplemented with garlic and apple pomace (3% w/w each, GA); and ETEC-challenged fed a diet supplemented with garlic and blackcurrant (3% w/w each, GB). Treatments were balanced by initial body weight. Feed and water were provided ad libitum*.* Diets followed the guidelines for organic pig production and were formulated to meet the Danish nutrient requirement standards for pigs [17].

The characteristics of garlic, apple pomace, and blackcurrant powders, as well as the ingredients and analyzed composition of the diets were as reported by Jerez-Bogota, et al. [8]. Feed formulation is presented Table S1, while analytical composition of the experimental diets is presented in Table S2. The processing conditions were considered to optimize the quantity of active compounds. Briefly, fresh organically cultivated garlic (Therador cultivar) and apple pomace from organic apples (Elstar cultivar) were processed into a fine powder (Ø = 0–1 mm). The garlic cloves were dried at 40 °C and the apple pomace at 60°C. Freeze-dried blackcurrant powder (Ø = 0–1 mm) from organic berries grown in Lithuania was sourced from Berrifine (Ringsted, Denmark). The allicin concentration of the garlic powder was 44.1 ± 6.0 mg alliin Eq/g dry powder. The pH of the apple pomace powder was 3.28 ± 0.01 and the pH of the blackcurrant powder was 2.81 ± 0.01.

The challenged groups were subjected to an ETEC F18 (strain 9,910,297-2STM) challenge as described by Jerez-Bogota, et al. [8]. Briefly, on days 1 and 2 after weaning, the challenged pigs received a 7–8 ml oral gavage having ~ 10^9^ colony forming units/ml of an overnight-grown ETEC F18 inoculum. One of the blocks presented an unusual ETEC shedding profile: i.e., rapid reduction in the ETEC-challenge treatments with a concomitant increase in the non-challenge treatment. The pigs from this block were therefore excluded from Jerez-Bogota, et al. [8], where the main focus was the ETEC course of infection. In the present study, however, both blocks were used for analyses, as we aimed to investigate the extended effects of plant material supplementation.

Piglets were in the experimental treatments for three weeks and were sampled immediately afterward at a time point when the acute phase of the ETEC challenge was expected to have resolved and the sustained effects of the antibacterial plant supplementation could be assessed. In two pens, both pigs died during the first week of the experiment (first block: GA; second block: PC).

In each block, one pig per pen was randomly selected for sample collection, resulting in 4 pigs per treatment per block (8 pigs per treatment), except for the two pens where early mortality reduced the number of pigs sampled to 7 in GA and PC. The total number of sampled pigs per treatment is indicated in all figure legends and table footnotes.

### Experimental sampling procedures

Three weeks after weaning, one pig per pen was randomly chosen and euthanized by captive bolt stunning followed by exsanguination. After exsanguination, the abdominal cavity was exposed, and the GIT was removed. The GIT was clamped to separate the stomach, small intestine, cecum, and colon, minimizing digesta movement. The small intestine and colon were each divided into three equal-length sections: proximal, middle, and distal. For simplicity, from now on in the text the proximal, middle, and distal small intestine sections are referred to as the duodenum, jejunum, and ileum, respectively (however not anatomically exact). The luminal contents (digesta) were squeezed from each of the eight sections: stomach, duodenum, jejunum, ileum, cecum, and colon (proximal, middle, distal). Digesta samples were collected in stomacher bags and stored at –20 °C for microbial metabolite analysis. Another digesta sample was collected in 2 ml microtubes, immediately snap-frozen in liquid nitrogen, and stored at −80 °C for genomic analyses. In the colon, only samples from the middle segment were used, and from now on, they are referred to as colon samples.

A section of the jejunum (50% small intestine length; 10 cm) and ileum segments (90% small intestine length; 10 cm) were opened longitudinally, the digesta was flushed with ice-cold phosphate-buffered saline, and the mucosa was scraped with sterile glass microscope slides. Peyer’s patches were avoided for Mucosal scrapping samples. Mucosal scrapings from the jejunum and ileum segments were divided into two subsamples in 2 ml Eppendorf tubes, snap-frozen in liquid nitrogen, and stored at − 80 °C for gene expression analysis and determination of antioxidant enzyme activity.

### Analytical methods

For laboratory analyses, the individual pig was considered the biological replicate, and one representative sample per pig was used. Laboratory analyses followed standard operating procedures, including two technical replicates per sample (with a coefficient of variation < 10%), except for qPCR assays, which were performed in triplicate.

The digesta pH was measured at the time of sampling via a calibrated digital pH meter (Meterlab PHM 220, Radiometer, Brønshøj, Denmark). The digesta DM was measured by freeze-drying a subsample to constant weight. Digesta samples from the stomach, jejunum, ileum, cecum and colon were used for genomic quantification of one of the F18 fimbriae genes (*fedA*), *E. coli*, *Lactobacillus* and total bacteria via quantitative PCR (qPCR). The DNA was extracted from digesta samples (Stomach: 200 mg, Jejunum and Ileum: 500 mg, Cecum and Colon: 50 mg) via the NucleoSpin 96 DNA Stool Kit (740,473.4, Macherey–Nagel, Germany), and the concentration of genomic double-stranded DNA was measured via the Qubit Broad Range Assay Kit (Thermo Fisher Scientific, Waltham, MA, USA) on an Invitrogen Qubit 4.0 Fluorometer (Thermo Fisher Scientific, Waltham, MA, USA). Following extraction, quantitative PCR was performed on a ViiA 7 real-time PCR system (Applied Biosystems, Waltham, MA, USA) using MicroAmp Optical 384 well reaction plates (Applied Biosystems, Waltham, MA, USA). The qPCR reactions included 5 μL of Maxima SYBR Green/ROX Master Mix (Thermo Scientific, Waltham, MA, USA), 0.3 + 0.3 μL of forward and reverse primers, 2.4 μL of nuclease-free H_2_O and 2 μL of cDNA. The samples were analyzed in triplicate, and the data were processed in QuantStudio (v1.4, Applied Biosystems, Waltham, MA, USA). The specific primers and qPCR settings are presented in Table 1.

**Table 1:** Primers used for quantitative PCR and settings for the quantification of selected bacterial groups

Target sequence	Sequence (5'−3')	Conc^1^ (µM)	TA^2^ (°C)	Size (bp)
F18 adheson (*fedA*)	F: GGAGGTTAAGGCGTCGAATAG	0.3	62	90
	R: CCACCTTTCAGTTGAGCAGTA			
Total *E. coli*	F: TGATTGGCAAAATCTGGCCG	0.5	65	211
	R: GAAATCGCCCAAATCGCCAT			
*Lactobacillus* (23S)	F: GCGGTGAAATTCCAAACG	0.3	60	216
	R: GGGACCTTAACTGGTGAT			
All bacteria (16S)	F: CGGYCCAGACTCCTACGG	0.3	65	200
	R: TTACCGCGGCTGCTGGCAC			

^1^Concentration

^2^Annealing temperature

Microbial metabolites (organic acids, biogenic amines, and indoles) were measured in digesta samples from the stomach, ileum, cecum and colon. The concentrations of organic acids (acetic, propionic, butyric, valeric, isobutyric, isovaleric, lactic and formic acids) were analyzed via capillary gas chromatography as described by Jensen, et al. [18], with modifications as described by Canibe, et al. [19]. Throughout the manuscript, organic acids are referred to in their protonated (acid) form for consistency, although in vivo their ionization state varies by gastrointestinal pH (e.g., acetic acid predominates in the stomach, while acetate is more prevalent in the intestine). The total concentration of short chain fatty acids (SCFA) was estimated as the sum of the concentrations of acetic, propionic, butyric and valeric acids, and individual SCFA were expressed as proportion of total SCFA for statistical analyses. The concentrations of indolic compounds (indole, indoleacetic acid, 3-indolepropionic acid and skatole) and p-cresol were analyzed via high-performance liquid chromatography (HPLC) as described by Knarreborg, et al. [20]. Biogenic amines (cadaverine, agmatine, putrescine and tyramine) were quantified by gradient elution via reversed-phase HPCL, as described by Poulsen, et al. [21]. The ammonia concentration in digesta was determined using a colorimetric method, using the chemical reaction of ammonia ions with sodium salicylate and nitroprusside in a buffer, the color reaction was measured at 650 nm wavelength in a microplate photometer (Multiskan CF, Thermo Scientific, Waltham, MA, USA).

Mucosal scrapings from the jejunum and ileum were analyzed for the concentrations of malondialdehyde (MDA) and the antioxidant enzymes superoxide dismutase (SOD) and glutathione peroxidase (GSH-Px). The samples were first homogenized in ice-cold PBS supplemented with 3 µl of protease inhibitor (Thermo Scientific, Waltham, MA, USA), followed by centrifugation at 1500 × g for 10 min at 4 °C; thereafter, the samples were analyzed using the appropriate assay kits. The SOD activity was measured with a colorimetric activity kit (K028-H, Arbor Assays, Ann Arbor, MI, USA), MDA activity was measured with a colorimetric detection kit (K077-H1, Arbor Assays, Ann Arbor, MI, USA), and GSH-Px activity was measured with a colorimetric activity kit (MAK437, Sigma‒Aldrich, Søborg, Denmark). Enzyme activity was expressed as units (**U**) per mg protein. The total protein content was measured using the Micro BCA Protein Assay kit (23,235, Applied Biosystems, Waltham, MA, USA).

Mucosal scrapings from the jejunum and ileum were analyzed for the gene expression of selected gut integrity indicator genes, namely, occludin (*OCLN*), zonula occludens protein-1 (*ZO-1*), and nuclear factor kappa B (*NF-κB*). Total RNA was isolated from jejunal and ileal mucosa samples via the NucleoSpin RNA Plus Kit (Macherey–Nagel, Düren, Germany). For extraction, 50 mg of mucosal sample was weighed and homogenized using a steel ball, and the remaining steps were performed according to the manufacturer’s instructions. The RNA quality was assessed in a Nanodrop spectrophotometer (Thermo Scientific, Waltham, MA, USA). Reverse transcription of the extracted RNA to cDNA was performed using the High-Capacity cDNA Reverse Transcription kit (Applied Biosystems, Waltham, MA, USA). Quantitative PCR was conducted on cDNA samples using the TaqMan Fast Advanced Master Mix (Applied Biosystems, Waltham, MA, USA) and TaqMan porcine probes. The PCR was performed on a thermocycler (ViiA7, Applied Biosystems, Waltham, MA, USA) with 2.0 µl of cDNA and three replicates per reaction, using a one-step PCR protocol (40 cycles, 95 °C—21 s and 60 °C—20 s) in assay volumes of 8 µl (5 µl of Mastermix, 0.5 µl of probe, and 2.5 µl of H_2_O). The software qbase + (Biogazelle NV, Gent, Belgium) was used to determine gene-specific amplification efficiencies by dilution series and to test for the expression stability of reference genes. The genes encoding for β-actin and glyceraldehyde 3-phosphate dehydrogenase were selected for normalization. After the normalization of Cq values with the respective reference genes, the results were scaled to the NC treatment as described by Taylor, et al. [22].

### Profiling of the gut microbiota

Digesta samples from the stomach, jejunum, ileum, cecum, and colon were used for microbiota profiling. The samples used for qPCR analyses were the same as those used for microbiota profiling; thus, the total DNA extraction method was as described above. All samples had DNA concentrations greater than 7 ng/µl and were used for PCR amplification and paired-end sequencing of the 16S rRNA gene V3‒V4 region (250 bp paired-end) on the Illumina NovaSeq 6000 platform (Illumina, San Diego CA, USA). Library preparation, DNA quality control (agarose gel electrophoresis; 5400 Fragment Analyzer, Agilent, Santa Clara, CA, USA), and sequencing were performed by Novogene UK (Cambridge, United Kingdom).

The raw amplicon sequencing data were processed into amplicon sequence variants (ASV) using the DADA2 pipeline [23] in R software (version 4.2). Reads were filtered and trimmed on the basis of quality, denoised, and merged, chimeras were removed, and taxonomy was assigned to each derived ASV using a naive Bayesian classifier method against the SILVA reference database v138 [24]. A phylogenetic tree was constructed using the FastTree2 algorithm [25].

Downstream analyses were performed in R (version 4.2.1). Subsequent filters included the removal of read lengths shorter than 398, non-bacterial and cyanobacteria reads. Analyses were performed after rarefying at 90% of the minimum sampling depth. The genus *Rhodoligotrophos* was detected in several stomach samples, particularly from the GA group. A BLAST search revealed that the *Rhodoligotrophos* sequences matched *Triticum aestivum* (wheat) mitochondrial DNA; thus, the reads were omitted from the diversity analyses. The α‒diversity (Observed, Shannon, and inverse Simpson) and β-diversity measures (weighted UniFrac dissimilarity matrices) were calculated via the *phyloseq* package. Differential abundance analysis was conducted via the *DEseq2* package, preceded by a filter of 0.1% relative abundance and 10% prevalence.

### Statistical analyses

The data from growth performance, fecal scores, qPCR enumeration of microbial groups, microbial metabolites, mucosal gene expression and mucosal antioxidant capacity were analyzed via the GLIMMIX procedure in SAS Studio (SAS Institute, Cary, NC, USA). The models included the effects of dietary treatment, the gut segment and their interaction as fixed effects, whereas pigs and blocks were included as random effects. Sow was included in the original model, but due to a lack of significance, it was removed from the final models. Adequate distributions and covariance structures were selected by best fit and regression diagnostics on conditional residuals. The *P*‒values were adjusted for multiple comparisons using the Holm‒Bonferroni method.

The 16S rRNA gene amplicon sequencing data analyses were performed in R (version 4.2.1). Microbiota α‒diversity was analyzed using generalized linear mixed models with dietary treatment, segment and interaction as fixed effects, and random effects of pigs and blocks. The effect of sow was not significant and was therefore removed from the final model. The models were fitted using the *glmmTMB* package, and adequate distributions and covariance structures were selected by best fit and regression diagnostics via the *DHARMa* package. The microbiota β‒diversity was examined by nonmetric multidimensional scaling (NMDS) ordination and compared using PERMANOVA in the *vegan* package [26]. Differentially abundant taxa were identified via the *DEseq2* workflow in two stages [27]. First, a likelihood ratio test was conducted using a model that included treatment, segment, and interaction. Each treatment within each segment was subsequently compared to the NC treatment using a pairwise Wald test. Differentially abundant taxa (log_2_ fold change > 2 and adjusted *P* < 0.05) were grouped using k‒means hierarchical clustering with the *pheatmap* package, the *P*‒values were adjusted for multiple comparisons using the Benjamini–Hochberg technique.

## Results

The PC group exhibited signs of diarrhea on days 7 and 9 post-weaning, as indicated by a mean fecal dry matter below 20%, the threshold for diarrhea (Figure S1). In contrast, the GA and GB groups maintained fecal dry matter percentages similar to the NC group, generally above 20%. By the time of sampling, three weeks after weaning and the ETEC challenge, pigs appeared healthy with no evident signs of diarrhea (according to health records; not shown). Moreover, all pigs exhibited similar growth performance (Table S3) and had comparable fecal dry matter at sampling (day 21 post-weaning), with mean values exceeding 25% (Figure S1).

The genomic counts of F18 fimbriae, *E. coli*, *Lactobacillus*, and total bacteria differed by segment (*P* < 0.001) but were unaffected by experimental treatment, regardless of the GIT segment (Table 2). From the 16S rRNA gene amplicon sequencing, an average of 1047 ASV were identified per sample from ~ 9.5 million reads. On average, 78% of the identified ASV were retained following size filtering, corresponding to 98.5% of the high-quality reads.

**Table 2:** Genomic quantification of selected bacteria in digesta along the gastrointestinal tract

Item	Treatment^1^				SEM^2^	*P* value^3^		
	NC	PC	GA	GB		Trt	Seg	Trt × Seg
F18 fimbriae						0.580	< 0.001	0.248
Stomach	2.88	3.30	3.30	3.17	0.159			
Jejunum	2.83	2.63	2.73	2.59	0.137			
Ileum	2.94	2.97	3.01	2.75	0.192			
Cecum	4.04	4.12	4.38	4.11	0.276			
Colon	3.47	3.99	4.06	3.84	0.204			
*E. coli*						0.718	< 0.001	0.650
Stomach	3.59	3.90	3.65	3.61	0.159			
Jejunum	3.92	3.75	3.29	3.75	0.137			
Ileum	4.53	4.54	4.22	4.29	0.192			
Cecum	5.79	5.77	5.78	5.69	0.276			
Colon	5.08	4.80	4.97	4.64	0.204			
*Lactobacillus*						0.835	< 0.001	0.628
Stomach	9.07	9.39	8.78	9.12	0.406			
Jejunum	8.76	8.66	8.42	8.82	0.287			
Ileum	9.25	9.27	8.95	9.31	0.167			
Cecum	10.13	9.88	9.99	10.19	0.126			
Colon	10.21	10.10	10.41	9.82	0.236			
All bacteria						0.855	< 0.001	0.667
Stomach	9.04	9.24	8.79	8.93	0.383			
Jejunum	8.59	8.44	8.51	8.69	0.312			
Ileum	9.52	9.63	9.35	9.65	0.162			
Cecum	10.81	10.73	10.83	10.88	0.101			
Colon	10.72	10.65	10.65	9.92	0.343			

^1^**NC**: non-challenged and organic diet (*n* = 8), **PC** ETEC-challenged and organic diet (*n* = 7); **GA**: ETEC-challenged, organic diet and garlic + apple pomace (3% + 3%; *n* = 7); **GB**: ETEC-challenged, organic diet and garlic + blackcurrant (3% + 3%; *n* = 8)

^2^Pooled standard error of least squared means

^3^**Seg**: Effect of gastrointestinal segment. **Trt**: effect of treatment **Trt** × **Seg**: interaction effect

**Fig. 1:**
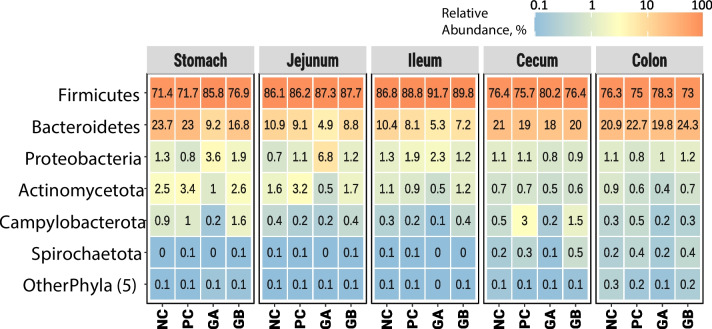
Relative abundance of bacterial phyla along segments of the gastrointestinal tract of pigs. The values indicate the mean relative abundance (%) of the six dominant phyla (Y-axis) across treatments (X-axis) and segments (panels). NC: non-challenged and organic diet (*n* = 8); PC: challenged and organic diet (*n* = 7); GA: challenged, organic diet and garlic + apple pomace (3% + 3%; *n* = 7); GB: challenged, organic diet and garlic + blackcurrant (3% + 3%; *n* = 8). Individual values are presented in Figure S2

**Fig. 2:**
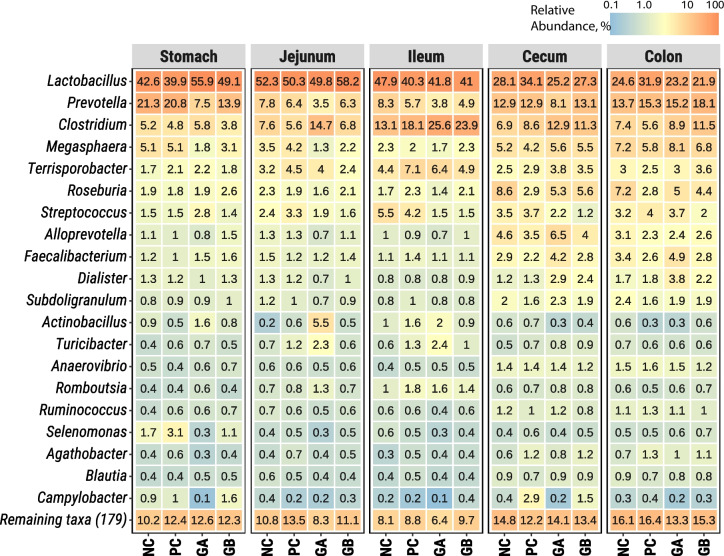
Relative abundance of bacterial genera along segments of the gastrointestinal tract of pigs. The values indicate the mean relative abundance (%) of the 20 dominant genera (Y-axis) across treatments (X-axis) and segments (panels). **NC**: non-challenged and organic diet (*n* = 8); **PC**: challenged and organic diet (*n* = 7); **GA**: challenged, organic diet and garlic + apple pomace (3% + 3%; *n* = 7); **GB**: challenged, organic diet and garlic + blackcurrant (3% + 3%; *n* = 8). Individual values are presented in Figure S3

The bacterial relative abundances by treatment and GIT segment are presented at the phylum level (Fig. 1: group means and Figure S2: individual values) and genus level (Fig. 2: group means and Figure S3: individual values). The most abundant phyla were Firmicutes (synonym Bacillota), Bacteroidetes (synonym Bacteroidota), and Proteobacteria (synonym Pseudomonadota). Firmicutes were the most abundant, exceeding 70% in all GIT segments and treatments. Bacteroidetes was the second most abundant phylum, particularly in the stomach, cecum, and colon (~ 20%), compared with lower levels in the jejunum and ileum (less than 11%). Proteobacteria were notably abundant in the stomach and jejunum in the GB treatment group.

The bacterial relative abundances by treatment and GIT segment are presented at the genus level (Fig. 2: group means and Figure S3: individual values). *Lactobacillus*, *Prevotella*, *Clostridium*, and *Megasphaera* accounted for approximately 70% of the relative composition in the stomach, jejunum, and ileum and 40–50% of the relative composition in the cecum and colon (Fig. 2 and Figure S3). In the cecum and colon, several other genera, e.g., *Roseburia*, *Streptococcus*, *Alloprevotella*, *Faecalibacterium*, and *Dialister*, had relatively low abundances. *Lactobacillus* was the most abundant genus, particularly in the stomach, jejunum, and ileum (> 40%). *Prevotella* was the second most abundant genus, particularly in the stomach, cecum, and colon, with similar abundances across the treatment groups (except for GA in the stomach and jejunum). *Clostridium* was particularly abundant in the ileum, where it was the second most abundant genus. *Clostridium* was more abundant (~ 25%) in the ileum under the GA and GB treatments than under the NC and PC treatments (~ 13% and ~ 18%, respectively). *Megasphaera* was particularly abundant in the cecum and colon in all the treatment groups. *Terrisporobacter* had similar relative abundances across GIT segments and treatments, except for the ileum, where it was more abundant than *Megasphaera* in all the treatments.

**Fig. 3:**
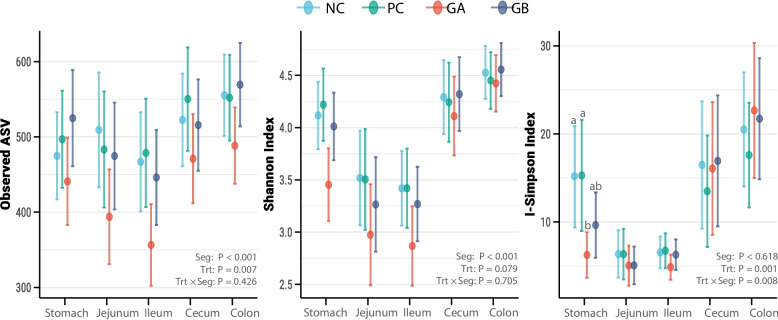
Measures of α diversity along segments of the gastrointestinal tract of pigs. Dots are least squared means, and lines are 95% confidence intervals. Seg: Effect of gastrointestinal segment. Trt: effect of treatment Trt × Seg: interaction effect. ^ab^ Values that do not share a common superscript differ (*P* < 0.05), Bonferroni–Holm adjustment. **NC**: non-challenged and organic diet (*n* = 8); **PC**: challenged and organic diet (*n* = 7); **GA**: challenged, organic diet and garlic + apple pomace (3% + 3%; *n* = 7); **GB**: challenged, organic diet and garlic + blackcurrant (3% + 3%; *n* = 8)

The treatments influenced (*P* = 0.007) the Observed α diversity regardless of the GIT segment (Fig. 3). The Observed α diversity of the GA treatment was lower than that of the other treatments (*P* < 0.05). The response of the Shannon index was similar to that of the Observed α diversity, but unaffected by treatment (*P* = 0.079).

**Fig. 4:**
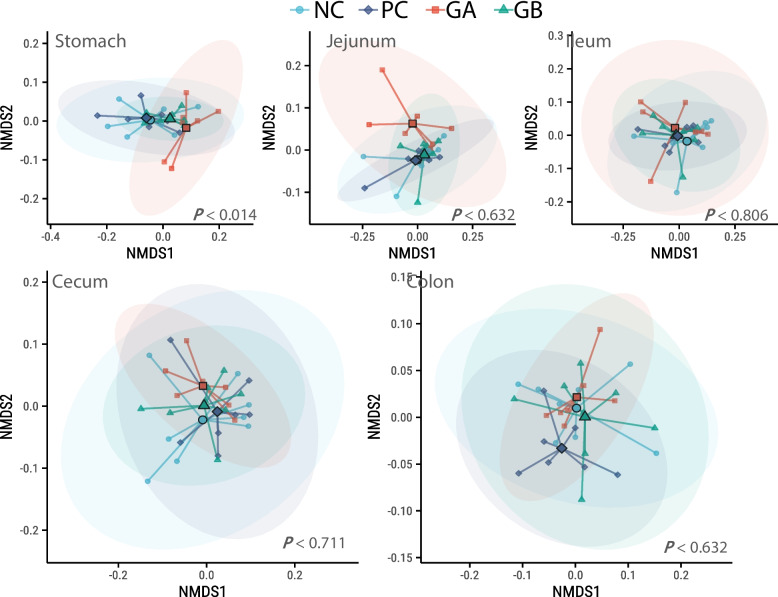
β diversity along segments of the gastrointestinal tract of pigs in the experimental treatments. Nonmetric multidimensional scaling (NMDS) plot of weighted UniFrac distances. The solid shapes in the ordination represent the mean centroids. **NC**: non-challenged and organic diet (*n* = 8); **PC**: challenged and organic diet (*n* = 7); **GA**: challenged, organic diet and garlic + apple pomace (3% + 3%; *n* = 7); **GB**: challenged, organic diet and garlic + blackcurrant (3% + 3%; *n* = 8)

Compared with the PC and NC treatments, the GA treatment had a lower (*P* < 0.05) α diversity in the stomach, as measured by inverse Simpson index. The effect of treatment on β-diversity was significant (*P* = 0.014) only in the stomach, where the GA and GB treatments tended to differ (*P* < 0.1) from the NC treatment (Fig. 4).

**Fig. 5:**
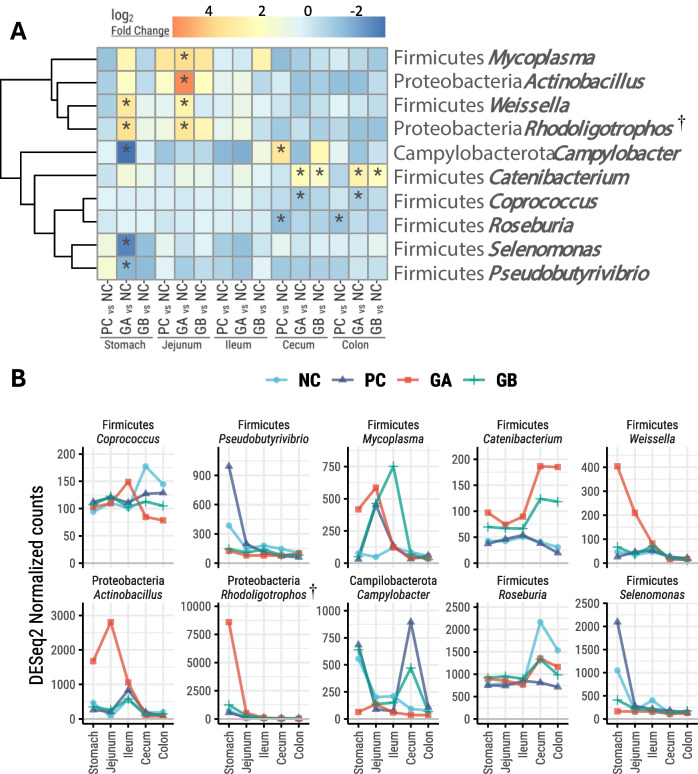
Differentially abundant bacterial genera along the gastrointestinal tract of the pigs. **NC**: non-challenged and organic diet (*n* = 8); **PC**: challenged and organic diet (*n* = 7); **GA**: challenged, organic diet and garlic + apple pomace (3% + 3%; *n* = 7); **GB**: challenged, organic diet and garlic + blackcurrant (3% + 3%; *n* = 8). **A**: Hierarchical cluster heatmap; color gradients represent log_2_-fold changes from NC. (*): *P* < 0.05 pairwise comparison against NC within the GIT segment. **B**: DESeq2-normalized counts of differentially abundant genera. (†): Misclassification of mitochondrial DNA (see main text)

The differential abundance analysis revealed that ten genera were significantly influenced by the treatment (log_2_-fold change > 2 and *P* < 0.05; Fig. 5). Compared with the NC, the GA treatment resulted in a greater abundance of *Weissella* in the stomach and *Mycoplasma*, *Actinobacillus*, and *Weissella* in the jejunum, as well as a lower abundance of *Campylobacter*, *Selenomonas*, and *Pseudobutyrivibrio* in the stomach. Compared with the NC treatment, the PC treatment resulted in a greater abundance of *Campylobacter* in the cecum and a lower abundance of *Roseburia* in the cecum and colon. Additionally, the GA and GB treatments resulted in a greater abundance of *Catenibacterium* in the cecum and colon than did the NC treatment. There was a lower abundance of *Coprococcus* in the ceca of pigs in the GA treatment than in those in the NC treatment.

**Fig. 6:**
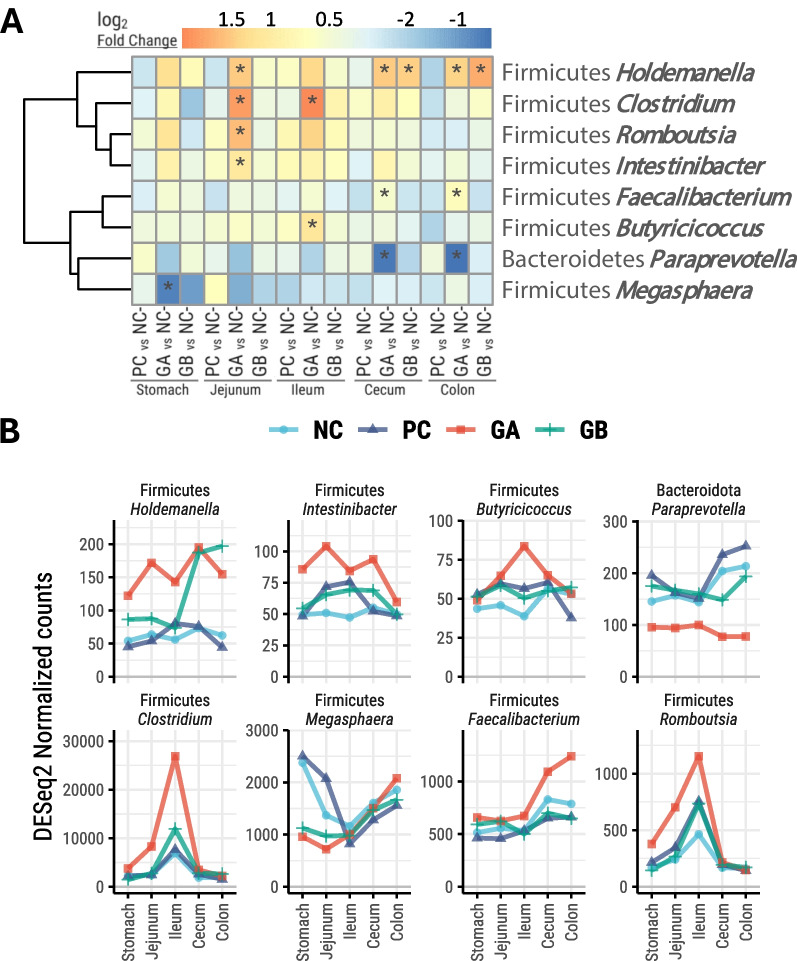
Non-differentially abundant genera influenced in the gastrointestinal tract of pigs. **NC**: non-challenged and organic diet (*n* = 8); **PC**: challenged and organic diet (*n* = 7); **GA**: challenged, organic diet and garlic + apple pomace (3% + 3%; *n* = 7); **GB**: challenged, organic diet and garlic + blackcurrant (3% + 3%; *n* = 8). **A** Hierarchical cluster heatmap; color gradients represent log_2_-fold changes from NC. (*): *P* < 0.05 pairwise comparison against NC within the GIT segment. **B** DESeq2-normalized counts of differentially abundant genera

The abundance of other bacteria was also significantly affected by the GA and GB treatments compared to the NC treatment, though the changes were of lower magnitude (*P* < 0.05 but log_2_ fold change < 2; Fig. 6). Compared to the NC treatment, the GA treatment reduced the abundance of *Megasphaera* in the stomach; increased *Intestinibacter*, *Romboutsia*, *Clostridium*, and *Holdemanella* in the jejunum; and increased *Clostridium* and *Butyricicoccus* in the ileum. Compared to the NC treatment, the GA and GB treatments had higher abundance of *Holdemanella* in the cecum and colon. Additionally, the GA treatment led to lower *Paraprevotella* and higher *Faecalibacterium* abundance in the cecum and colon compared to the NC treatment. The overall normalized counts of the genera used for differential abundance analyses are presented in Figures S2 and S3.

In the stomach, the GA treatment resulted in the lowest (*P* < 0.05) digesta DM (Table 3). Across all GIT segments, lactic acid concentrations were lower (*P* < 0.05) in the GA and GB treatments than in the PC treatment; however, lactic acid concentrations were lower in the GA treatment than in the NC treatment. Although the concentration of total SCFA did not differ in the stomach, the GA and GB treatments resulted in a greater proportion of acetic acid and a lower proportion of propionic acid than did the PC treatment. In the cecum, the SCFA concentration in the GB treatment was greater than that in the NC and PC treatments, and in the colon, it was greater than that in the NC and PC treatments. Additionally, in the colon, the proportion of butyric acid was greater in the GA and GB treatment groups than in the NC treatment group. The digesta pH and formic acid, valeric acid and BCFA concentrations were not affected (*P* > 0.05) by treatment.

**Table 3:** Digesta dry matter (DM), pH, and concentrations of organic acids along the gastrointestinal tract

Item^2^	Treatment^1^					*P* value^4^		
	NC	PC	GA	GB	SEM^3^	Trt	Seg	Trt × Seg
DM, %						0.639	< 0.001	0.005
Stomach	27.1^a^	27.4^a^	22.2^b^	26.21^a^	1.15			
Ileum	13.3	12.9	13.7	12.68	1.15			
Cecum	12.9	14.1	15.6	13.39	1.15			
Colon	20.8	23.1	23.3	22.05	1.16			
pH						0.272	< 0.001	0.994
Stomach	2.9	2.7	3.1	2.96	0.23			
Ileum	6.6	6.4	6.9	6.48	0.23			
Cecum	5.7	5.7	5.9	5.79	0.23			
Colon	6.2	6.1	6.2	6	0.23			
Formic acid, mmol/kg						0.077	< 0.001	0.235
Stomach	0.4	0.3	0.5	0.26	0.21			
Ileum	4.9	8.7	9.4	6.78	1.47			
Cecum	0.4	1.0	0.4	0.33	0.3			
Lactic acid, mmol/kg	_ab_	_a_	_c_	_bc_		< 0.001	< 0.001	0.208
Stomach	17.3	21.3	6.9	16.1	0.37			
Ileum	32.6	34.1	12.7	36.0	0.37			
Cecum	5.8	13.2	1.7	2.0	0.46			
SCFA, mol/kg						0.034	< 0.001	0.031
Stomach	14.1	16.8	17.4	14.3	4.79			
Ileum	6.1	8.6	8.4	7.3	4.79			
Cecum	107.7^b^	106.4^b^	114.6^ab^	124.1^a^	4.79			
Colon	113.7^b^	125.6^ab^	124.3^ab^	135.8^a^	4.79			
Acetic acid, %						0.278	< 0.001	0.014
Stomach	70.43^b^	66.60^b^	83.23^a^	79.59^a^	2.97			
Ileum	93.7	95.21	95.94	94.28	2.97			
Cecum	58.72	59.73	58.07	57.04	2.97			
Colon	57.95	56.63	55.32	54.77	2.98			
Propionic acid, %						0.027	< 0.001	0.005
Stomach	20.66^b^	27.39^a^	11.07^c^	15.29^bc^	2.03			
Ileum	2.88	2.36	1.85	2.67	2.03			
Cecum	29.64	27.51	27.86	28.32	2.03			
Colon	25.83	24.97	25.23	24.75	2.06			
Butyric acid, %						0.532	< 0.001	0.032
Stomach	6.9	4.8	4	3.9	1.16			
Ileum	2.2	1.8	1.8	2.4	1.16			
Cecum	10.1	11	12.3	12.5	0.87			
Colon	13.2^b^	15.1^ab^	16.4^a^	17.0^a^	0.87			
Valeric acid, %						0.759	< 0.001	0.661
Stomach	2.1	1.3	1.9	1.3	0.49			
Ileum	1.3	0.7	0.7	0.8	0.49			
Cecum	1.7	1.8	2.1	2.2	0.49			
Colon	3.1	3.2	3.3	3.6	0.49			
Iso-acids, mol/kg						0.759	< 0.001	0.661
Stomach	2.1	1.3	1.9	1.3	0.48			
Ileum	1.2	0.7	0.7	0.7	0.48			
Cecum	1.6	1.8	2.0	2.2	0.48			
Colon	3.1	3.2	3.2	3.5	0.48			

^1^ *NC* non-challenged and organic diet (*n* = 8); *PC* ETEC-challenged and organic diet (*n* = 7); *GA *ETEC-challenged, organic diet and garlic + apple pomace (3% + 3%; *n* = 7); *GB* ETEC-challenged, organic diet and garlic + blackcurrant (3% + 3%; *n* = 8)

^2^ Organic acids are listed in their protonated (acid) form for consistency, although their ionization state varies across the gastrointestinal tract depending on local pH and the compound’s pKa. For example, acetic acid (pKa ≈ 4.76) is mostly undissociated in the stomach (pH ~ 3), but largely dissociated to acetate in the intestine (pH ~ 6–7). *SCFA* short-chain fatty acids (acetic, propionic, butyric, valeric acids).* Iso-acids* isobutyric and isovaleric acids. Detection levels (mmol/kg): Formic acid = 0.5, acetic acid = 0.4, propionic acid = 0.2, isobutyric acid = 0.1, butyric acid 0.1, isovaleric acid = 0.1, valeric acid = 0.1, and DL-lactic acid 0.6

^3^ Pooled standard error of least squared means

^4^*Seg *Effect of gastrointestinal segment, *Trt* effect of treatment, *Trt* × *Seg* interaction effect

^abc^ Within a row, values that do not share a common superscript differ (*P* < 0.05), Bonferroni–Holm adjustment. When interaction is not significant, the letters represent differences among the treatments, regardless of the segment

Indole, indoleacetic acid, indol-3-propionic acid, and p-cresol were below the detection limits in the stomach and ileum samples (Table 4). In the cecum, the concentration of skatole in the GB treatment was lower (*P* < 0.05) than that of the NC and PC treatments, whereas in the colon, the indole concentration of the GA treatment was lower (*P* < 0.05) than that of the GB treatment. The concentration of 3-indolepropionic acid of the GB treatment was higher (*P* < 0.05) than that of the NC, PC and GA treatments, whereas the concentration of p-cresol in the GB treatment was lower than that in the PC treatment.

**Table 4:** Digesta concentration of indolic metabolites along the gastrointestinal tract

Item, mg/kg^2^	Treatment^1^				SEM^3^	*P* value^4^		
	NC	PC	GA	GB		Trt	Seg	Trt × Seg
Indole						0.169	< 0.001	0.047
Cecum	2.16	2.16	3.46	4.13	0.535			
Colon	4.83^ab^	6.65^ab^	3.26^b^	7.27^a^	0.991			
Indoleacetic acid						0.084	0.002	0.218
Cecum	8.12	5.68	10.07	15.23	1.494			
Colon	2.01	2.15	3.25	2.74	0.396			
3-indolepropionic acid	_b_	_b_	_b_	_a_		0.007	0.278	0.916
Cecum	1.01	1.11	1.35	1.9	0.417			
Colon	1.19	1.37	1.54	1.89	0.464			
p-cresol	_b_	_a_	_ab_	_b_		< 0.001	< 0.001	0.178
Cecum	1.66	1.92	2.01	1.55	0.148			
Colon	3.57	4.0	3.29	3.95	0.283			
Skatole						0.623	< 0.001	0.034
Stomach	0.8	0.54	0.97	0.64	0.163			
Ileum	0.88	0.59	0.90	0.92	0.209			
Cecum	6.79^a^	6.33^a^	4.13^ab^	2.72^b^	1.900			
Colon	28.22	30.39	21.81	28.31	4.883			

^1^ **NC**: non-challenged and organic diet (*n* = 8); **PC**: challenged and organic diet (*n* = 7); **GA**: challenged, organic diet and garlic + apple pomace (3% + 3%; *n* = 7); **GB**: challenged, organic diet and garlic + blackcurrant (3% + 3%; *n* = 8)

^2^ Detection levels (ppm): indol-3-acetic acid = 0.3, indol-3-propionic acid = 0.2, 4-methylphenol (p-cresol) = 1.9, 1-benzopyrrol (indole) = 0.1, 3-methylindole (skatole) = 0.1. Only skatole levels were above the detection level in stomach and ileum samples

^3^ Pooled standard error of least squared means

^4^ **Seg**: Effect of gastrointestinal segment. **Trt**: effect of treatment. **Trt** × **Seg**: interaction effect

^abc^ Within a row, values that do not share a common superscript differ (*P* < 0.05), Bonferroni–Holm adjustment. When there is a nonsignificant interaction, the letters stand for differences among the treatments, regardless of the segment

The ammonia concentration in digesta was influenced in the cecum and colon (Table 5). In the cecum and colon, the ammonia levels in the GA treatment were lower (*P* < 0.05) compared to the NC. The ammonia levels in the colon of the GA treatment were also lower (*P* < 0.05) that in the PC treatment. In the cecum, the putrescine concentration in the GB treatment was higher (*P* < 0.05) than in the PC and GA treatments, while the concentration in the GA treatment was lower (*P* < 0.05) than in the PC treatment. In the colon, the concentration of putrescine in the GA treatment was greater (*P* < 0.05) than that in the PC treatment. Cadaverine levels were lowest in the GA treatment in both the stomach and ileum, significantly different from those in the NC and PC treatments in the stomach and from those in the NC treatment in the ileum.

**Table 5:** Digesta concentrations of ammonia and biogenic amines along the gastrointestinal tract

Item, mg/kg^2^	Treatment^1^				SEM^3^	*P* value^4^		
	NC	PC	GA	GB		Trt	Seg	Trt × Seg
Ammonia						0.017	< 0.001	0.027
Stomach	2.71	2.95	2.67	3.34	0.685			
Ileum	5.24	7.38	6.04	6.19	0.765			
Cecum	8.00^a^	7.32^ab^	5.16^b^	6.64^ab^	0.805			
Colon	11.00^a^	12.59^a^	8.10^b^	10.16^ab^	0.776			
Agmatine						0.260	< 0.001	0.854
Stomach	11.44	9.97	11.85	11.12	2.532			
Ileum	15.66	14.42	20.57	16.1	3.761			
Cecum	7.51	5.53	6.36	7.53	0.779			
Colon	7.47	7.14	9.9	9.31	1.085			
Putrescine								
Stomach	18.95	22.01	10.65	20.68	5.566	0.343	< 0.001	0.0216
Ileum	17.9	13.84	8.33	16.17	3.638			
Cecum	92.45^a^	60.17^b^	59.98^b^	92.80^a^	14.845			
Colon	36.31^ab^	32.92^b^	56.34^a^	42.82^ab^	7.233			
Cadaverine						0.099	< 0.001	0.036
Stomach	34.15^a^	34.96^a^	10.67^b^	24.88^ab^	8.369			
Ileum	32.97^a^	22.65^ab^	10.54^b^	19.98^ab^	5.658			
Cecum	89.98	58.38	43.5	67.14	14.764			
Colon	116.66	54.33	72.36	58.14	23.234			
Tyramine						0.212	0.133	0.948
Stomach	7.13	5.88	5.63	8.04	2.112			
Cecum	4.51	4.44	5.24	8.12	4.244			
Colon	3.04	3.49	4.4	4.83	2.185			

^1^ **NC**: non-challenged and organic diet (*n* = 8); **PC**: challenged and organic diet (*n* = 7); **GA**: challenged, organic diet and garlic + apple pomace (3% + 3%; *n* = 7); **GB**: challenged, organic diet and garlic + blackcurrant (3% + 3%; *n* = 8)

^2^ Detection levels (mg/kg): Agmatine = 4.6, putrescine = 1.6, cadaverine = 1.8, and tyramine = 1.9

^3^ Pooled standard error of least squared means

^4^ **Seg**: Effect of gastrointestinal segment. **Trt**: effect of treatment. **Trt** × **Seg**: interaction effect

^abc^ Within a row, values that do not share a common superscript differ (*P* < 0.05), Bonferroni–Holm adjustment

The mucosal SOD and GSH-PX levels in the GA and GB treatments were greater (*P* < 0.05) than those in the NC and PC treatments, except for the GSH-PX values in the GB treatment, which did not differ significantly from those in the PC treatment (Table 6). Although the treatment did not significantly influence the mucosal MDA content (*P* = 0.0516), the mean values for the PC treatment in the ileum were numerically greater than those of the other treatments. The mucosal expression of the *OCLN*, *ZO-1* and *NF-κB* genes was not influenced by treatment.

**Table 6:** Small intestinal mucosal superoxide dismutase (SOD), glutathione peroxidase (GSH-Px), malondialdehyde (MDA) and integrity gene expression

Item^2^	Treatment^1^				SEM^3^	*P* value^4^		
	NC	PC	GA	GB		Trt	Seg	Trt × Seg
*Antioxidant activity*								
SOD, U/mg prot	_b_	_b_	_a_	_a_		< 0.001	0.874	0.712
Jejunum	71.3	79.5	90.08	92.26	6.09			
Ileum	76.69	76.4	90.58	85.68	6.018			
GSH-Px, U/mg prot	_b_	_b_	_a_	_ab_		< 0.001	0.996	0.739
Jejunum	78.66	85.67	102.66	101.78	6.602			
Ileum	84.89	86.75	101.4	94.57	6.701			
MDA, mmol/mg prot						0.0516	0.222	0.472
Jejunum	0.71	0.75	0.66	0.63	0.079			
Ileum	0.74	0.98	0.65	0.71	0.089			
*Gene Expression*								
* OCLN*, fold change						0.989	0.783	0.847
Jejunum	1.03	1.18	1.01	1.14	0.221			
Ileum	1.01	1.12	1.13	0.96	0.258			
*ZO-1*, fold change						0.700	0.930	0.830
Jejunum	1.03	1.21	1.29	1.19	0.194			
Ileum	1.01	1.32	1.15	1.21	0.191			
*NF-κB*, fold change						0.703	0.388	0.410
Jejunum	1.05	1.42	1.79	1.20	0.305			
Ileum	1.02	1.46	1.03	1.25	0.302			

^1^***OCLN***: Occludin, ***ZO-1***: Zonula occludens 1, ***NF-κB***: nuclear factor kappa B, gene expression relative to NC treatment

^2^**NC**: nonchallenged and organic diet (*n* = 8); **PC**: challenged and organic diet (*n* = 7); **GA**: challenged, organic diet and garlic + apple pomace (3% + 3%; *n* = 7); **GB**: challenged, organic diet and garlic + blackcurrant (3% + 3%; *n* = 8)

^3^ Pooled standard error of least squared means

^4^ Seg: Effect of gastrointestinal segment. Trt: effect of treatment. Trt × Seg: interaction effect

^abc^ Within a row, values that do not share a common superscript differ (*P* < 0.05), Bonferroni–Holm adjustment. When there is a nonsignificant interaction, the letters stand for differences among the treatments, regardless of the segment

## Discussion

In a previous study, we reported that garlic combined with either apple pomace or blackcurrant effectively prevented PWD caused by ETEC and alleviated the associated disruption to the fecal microbiota, particularly during the first week post-weaning [8]. Building on these findings, the current study aimed to evaluate the extended impact of these antibacterial plant combinations on the gut microbiota, microbial metabolites, intestinal integrity, and antioxidant activity at a later stage in the postweaning period.

In the current study, we collected samples three weeks after weaning—two weeks after the peak of ETEC infection—at a time when clinical signs had resolved and acute microbiota disturbances were expected to have subsided. This timing allowed us to evaluate the residual effects of a transient dietary intervention during the nursery phase, which typically spans the first 3–4 weeks after weaning in pig production systems. Several bioactive compounds in the supplemented plant materials, when unabsorbed and transiting through the gastrointestinal tract, can interact with gut microorganisms and exert local prebiotic, anti-inflammatory, and antioxidant effects [28]. Meanwhile, ETEC infection at weaning disrupts the developing microbiome, with consequences that may persist beyond the acute phase [29, 30]. The selected time point thus enabled us to assess both the lasting effects of ETEC challenge and the modulatory impact of the antibacterial plant supplementation. While later sampling could reveal long-term microbiome changes, this study specifically targeted the immediate postweaning period relevant for practical postweaning interventions.

Comparing the NC (non-challenged) and PC (ETEC-challenged) treatments in the current study provides a distinction between the effects of the weaning ETEC challenge and the contributions of antibacterial plant supplementation during the postweaning phase. As previously noted, the NC group in one of the experimental blocks exhibit an unusual response in ETEC shedding. However, fecal dry matter values in NC pigs remained stable and non-diarrheic, showing minimal influence from block effects. These observations reinforce our classification of the NC group as non-challenged. Despite strict biosecurity protocols, incidental ETEC exposure (experimental or environmental) cannot be fully excluded. Nevertheless, the absence of clinical signs and distinct microbial profiles in NC pigs supports this classification. Including both experimental blocks did introduce some variability but provided a more realistic reflection of practical production conditions. Growth performance data were included descriptively; however, these metrics were not the primary study focus and thus should be interpreted with caution.

Three weeks after weaning, the absolute numbers of F18 fimbriae, *E. coli*, *Lactobacillus*, and total bacteria along the GIT were unaffected by the ETEC F18 challenge at weaning or the dietary supplementation with the antibacterial plant combinations, as measured by qPCR. This finding aligns with our previous report, which showed that the impact on major infection indicators, including ETEC shedding, diarrhea, and growth depression, was most pronounced during the first week after weaning and had normalized by three weeks [8]. Nonetheless, lasting modulation of the gut microbiota by weaning ETEC exposure [31], and the intake of garlic [32], apple pomace [11, 33] or blackcurrant [34] have been reported in pigs and other species. In the present study, 16S rRNA gene sequencing revealed that both the ETEC challenge and plant supplementation influenced bacterial profiles throughout the GIT. This indicates that microbiota modulation occurred despite the unchanged absolute numbers of *E. coli* (including F18 fimbriae genes), *Lactobacillus*, and total bacteria.

The PC and NC treatments had generally similar bacterial profiles throughout the GIT, as reflected by their similar α- and β-diversities. *Lactobacillus* numbers and lactic acid were both high in the PC and NC treatments. Although the stomach is not the primary site for enteric fermentation, in growing pigs developing their gastrointestinal function, carbohydrate fermentation can be initiated by gastric microbes, resulting in relatively high SCFA and lactic acid contents [35]. Thus, the *Lactobacillus* numbers in the stomach could result from the gradual increase in feed intake after weaning [1] and the dietary inclusion of barley and oats (high in β-glucans), which are known to increase the gastric concentrations of *Lactobacillus* and β-glucan-degrading organisms [36]. Moreover, β-Glucans increase viscosity, which in turn increases retention time [36] and favors the presence of complex carbohydrate-degrading bacteria in the stomach [37]. Indeed, the 16S rRNA gene sequencing results in the present study revealed that *Lactobacillus*, *Prevotella*, *Clostridium* and *Megasphaera* were the most abundant genera in the gastric microbiota. Notably, we further observed an increased proportion of gastric propionate in the pigs from the PC treatment. Lactic acid is metabolized to propionate by bacteria such as *Selenomonas* and *Megasphaera* [38] which were also present at high relative abundance in the PC treatment.

In the large intestine, the PC treatment showed a lower abundance of *Roseburia* compared to the NC treatment. *Roseburia* is a known polyamine producer genus [39], which could explain the comparatively reduced cecal concentrations of putrescine in the PC treatment, as both the NC and PC pigs were fed the same diet. The role of putrescine and other biogenic amines in intestinal health is diverse. While excessive amounts are toxic, appropriate levels help support intestinal function and enhance immunity [5, 39]. Interestingly, the lower abundance of *Roseburia* in the large intestine in the PC treatment group than in the NC group was concomitant with a greater abundance of *Campylobacter*. *C. jejuni* and *C. coli* are considered zoonotic bacteria, with *C. coli* being the main *Campylobacter* species colonizing the pig GIT [40]. In contrast, *Roseburia* is often associated with gastrointestinal health [41]. Although this inverse relationship between *Roseburia* and *Campylobacter* has been observed in previous pig studies [42–44], a direct interaction between these genera has not yet been characterized and warrants further investigation.

Overall, our findings suggest that ETEC exposure at weaning may favor the persistence of potential pathogens in the gut at the expense of beneficial taxa. Previous studies have reported ETEC-induced shifts in the microbiota, including fecal changes at day 36 [45] and cecal alterations at day 21 post-weaning [32]. However, most ETEC challenge studies focus on microbiota profiles within one or two weeks after challenge [46], with few extending beyond this period, complicating comparisons.

Compared with the control treatment, garlic and apple pomace (GA treatment) had distinct effects on the upper GIT of the pigs. Notably, GA resulted in lower gastric DM and lower concentrations of lactic acid and cadaverine while maintaining similar SCFA concentrations, but with a greater proportion of acetic acid. The changes on microbial metabolites concentration in GA suggests a distinct shift in microbial metabolism likely driven by the selective effects of the garlic and apple pomace supplementation on the gastric microbiome. Garlic contains several antimicrobial compounds (e.g. allicin and other organosulfur compounds), that may reduce the abundance of sensitive taxa. Meanwhile, fructans from garlic along with polyphenols and fermentable fibers present in apple pomace could support the growth of specific microbial populations.

The observed changes in microbial metabolites of the GA were accompanied by a reduction in α diversity, particularly in the upper GIT. Although reduced α diversity is often associated with dysbiosis (or antibiotic usage [47]), in this case, the values remained within the range reported for healthy pigs [48, 49], and no signs of intestinal dysfunction were noted. Therefore, the reduced α diversity in GA likely reflects selective microbial modulation rather than compromised microbial health. Taken together, GA diet exerted a targeted effect on the upper gut microbiota, modulating bacterial composition and metabolism without inducing dysbiosis.

We observed that the GA had a greater relative abundance of the *Weissella* genus, in comparison to the control group. *Weissella* is genus that includes several heterofermentative probiotic strains with lactic and acetic acid as end products [50], and known for being resistant to garlic, making it the predominant in garlic-containing fermented products [50, 51]. Interestingly, this increase was not observed in the GB treatment, which also contained garlic. The discrepancy could be due to the differing bioactive profiles of apple pomace and blackcurrant. While apple pomace provides fermentable fibers and polyphenols that may synergize with garlic to promote *Weissella*, blackcurrant’s higher anthocyanin content may modulate microbial dynamics differently.

In addition to *Weissella*, the upper GIT of the GA treatment group also presented increased abundances of other genera, including *Holdemanella, Clostridium, Romboutsia, Intestinibacter*, and *Butyricicoccus*. Bacteria in these genera are generally commensal bacteria typically found in the pig gut and are associated with carbohydrate fermentation and bile acid metabolism [52–55]. In growing pigs, protein fermentation in the upper gut (stomach or small intestine) is undesirable, reducing protein availability for lean tissue growth, increasing inflammation, and potentially causing dysbiosis. The relatively high CP content of the diets in the current study, which is typical of organic diets, could promote protein fermentation [2, 56]. However, the inclusion of fermentable carbohydrates may mitigate this effect by favoring the growth of fibrolytic bacteria [56, 57]. Notably, the GA treatment reduced cadaverine levels in the stomach and ileum, and ammonia levels in the cecum and colon, which indicates decreased protein fermentation. However, the concentrations of isobutyric and isovaleric acids, derived from branched-chain amino acid fermentation, were not influenced by the treatments. Given the similar CP and total dietary fiber contents across all the diets, we hypothesize that the differences in protein fermentation metabolites may result from the combined antibacterial and prebiotic effects of the GA treatment. Nonetheless, we acknowledge that this interpretation warrants further validation through targeted mechanistic studies.

The GA treatment also increased the abundances of *Actinobacillus* and *Rhodoligotrophos* in the jejunum. In the present study, *Rhodoligotrophos* was a misclassification of mitochondrial DNA. A BLAST search of the *Rhodoligotrophos* sequences matched *Triticum aestivum* (wheat) mitochondrial DNA, indicating a dietary origin. Recently, it has been suggested that marine bacteria, such as *Rhodoligotrophos*, are the closest bacteria to mitochondria [58], which could explain the misclassification. While some *Actinobacillus* strains are respiratory pathogens, others are common inhabitants of the pig upper GIT and have been shown to increase with high-protein diets, low residual feed intake, and leanness [49, 53, 59]. *Campylobacter,* which includes some pathogenic strains (as mentioned earlier), was reduced by the GA treatment. The *Campylobacter* reduction may indicate bacterial susceptibility to the garlic and apple pomace combination or result from competitive exclusion by other commensal bacteria promoted by the GA treatment.

The effects in the large intestine were more similar between the GA and GB treatments. In the cecum and colon, the GA and GB treatments increased the abundance of *Catenibacterium,* a known SCFA producer [60]. Indeed, *Catenibacterium* has been found in greater abundance in the gut of pigs receiving oat bran [61] and apple pomace supplementation [11]. Pigs in the GA and GB treatments also presented increased relative abundances of *Holdemanella* and the GA of *Faecalibacterium*. Additionally, both the GA and GB treatments resulted in higher SCFA concentrations in the large intestine, notably in the cecum, with a slight increase in butyric acid proportions, which could be linked to the proliferation of butyrate-producing species (e.g., *Faecalibacterium prausnitzii*) [62]. Among SCFA, butyric acid is considered important for intestinal health because of its role as an energy source for colonocytes, anti-inflammatory effects, and ability to influence the expression of genes related to cell proliferation and apoptosis [62].

Dietary supplementation with garlic and either apple pomace or blackcurrant differently influenced putrescine and cadaverine levels in the large intestine. As mentioned above, moderate levels of some biogenic amines have beneficial effects on gut health [63, 64]. The levels of putrescine and cadaverine observed in the present study were lower than those reported to cause adverse effects on colonocytes in vitro, specifically 5 mM (440.75 mg/kg) putrescine and 2.5 mM (255.45 mg/kg) cadaverine [65]. Furthermore, we did not observe changes in the gene expression of selected gut barrier integrity markers (i.e. OCLN, ZO-1 genes), indicating that the pigs were not experiencing gastrointestinal injury. The specific impacts of putrescine and cadaverine on the gut may differ, with cadaverine potentially having more detrimental effects at lower concentrations [65]. On the other hand, putrescine has been linked to various virulence factors and disease-related phenotypes in both Gram + and Gram- bacteria [66]. Thus, the differential modulation of putrescine and cadaverine by GA and GB warrants further study.

Regardless of dietary fiber levels, diets high in CP (as in the present study) can moderately increase oxidative stress [57] creating an imbalance between reactive oxygen species and antioxidant defenses that can potentially damage cellular components such as lipids, proteins, and DNA, potentially contributing to inflammation [62]. The enzymes SOD and GSH-Px are key antioxidants that neutralize free oxygen radicals and reduce oxidative damage in various body tissues, including the intestinal mucosa [67, 68]. In the current study, pigs receiving GA or GB supplementation presented increased SOD and GSH-Px mucosal concentrations. Additionally, the PC treatment resulted in greater MDA, a common marker of lipid peroxidation [69]. The intake of phytochemicals has been shown to exert antioxidant effects and enhance the endogenous antioxidant system, potentially through direct interactions with molecular targets or by influencing metabolic pathways [70]. Furthermore, the promotion of bacteria associated with antioxidant activity has also been proven to be beneficial for the oxidative status of piglets [71]. For instance, some lactic acid bacteria can ease oxidative stress by producing enzymatic and non-enzymatic antioxidants, thereby protecting against the cellular damage caused by reactive oxygen species [72]. Thus, the increased activity of the antioxidative enzymes observed in the current study could be due to the indirect effects of plant phytochemicals present in garlic, apple pomace, and/or blackcurrant or to the favorable effects on specific bacterial strains with antioxidative activity.

The NF-κB signaling pathway is involved in oxidative stress responses and the development and progression of inflammation [62], but its specific role in the present study was not evident. No significant differences were observed in NF-κB expression between treatment groups, nor in the expression of tight junction genes, *OCLN* and *ZO-1*, which further indicates that the inflammatory response triggered by the ETEC challenge was resolved by three weeks after weaning (and ETEC infection). Although no direct evidence of NF-κB inhibition was observed, phytochemicals are known to modulate inflammatory pathways, including NF-κB activation [9, 73], and may have contributed to the recovery process during earlier stages of infection.

Overall, while earlier ETEC exposure did not drastically alter the overall gut microbiota composition, it induced shifts in specific bacteria, favoring potential pathogens such as *Campylobacter* and reducing beneficial bacteria such as *Roseburia*. In contrast, dietary supplementation with garlic and apple pomace modulated the upper gastrointestinal tract ecosystem, increasing the abundance of beneficial bacteria and reducing protein fermentation. Both garlic combined with either apple pomace or blackcurrant positively influenced the large intestine microbial ecosystem, increasing SCFA production, suggesting a role in promoting overall gut health. Other studies have shown that constant exposure to garlic powder can inhibit gut pathogens without significantly reducing the number of commensal bacteria [74, 75]. Notably, the combination with apple pomace appeared to have a more pronounced effect than blackcurrant, suggesting differential action by specific phytochemicals in each plant material, which warrants further investigation.

## Conclusion

The ETEC exposure at weaning did not drastically alter the overall pig gut microbiota composition after three weeks but resulted in an increased abundance of potential pathogens such as *Campylobacter* and a reduction in beneficial bacteria such as *Roseburia*. Dietary supplementation with garlic and apple pomace or blackcurrant modulated the gut microbiota, microbial metabolic activity, and antioxidant responses in weaned pigs after three weeks of supplementation, with GA primarily influencing the stomach and small intestine and both GA and GB impacting the cecum and colon, potentially offering protection against oxidative stress and favoring complex carbohydrate fermentation. Further research into the specific mechanisms underlying these effects, with a focus on the differential interactions of apple pomace and blackcurrant with garlic phytochemicals, could be of interest.

## Supplementary Information

Supplementary Material 1.

Supplementary Material 2.

Supplementary Material 3.

Supplementary Material 4.

Supplementary Material 5.

Supplementary Material 6.

## Data Availability

The data for this study have been deposited in the European Nucleotide Archive (ENA) at EMBL-EBI under accession number PRJEB77904 (https://www.ebi.ac.uk/ena/browser/view/PRJEB77904).
