# Supplementary figures and images for: Effects of supplementation of garlic with apple pomace or blackcurrant on the gastrointestinal microbial ecosystem of organic pigs after weaning
Source: BMC Microbiol. 2025 Oct 2;25:608. doi: 10.1186/s12866-025-04247-2 (PMC12492707; doi:10.1186/s12866-025-04247-2)

# LS-Means for Day\*Treatment

With 95% Confidence Limits

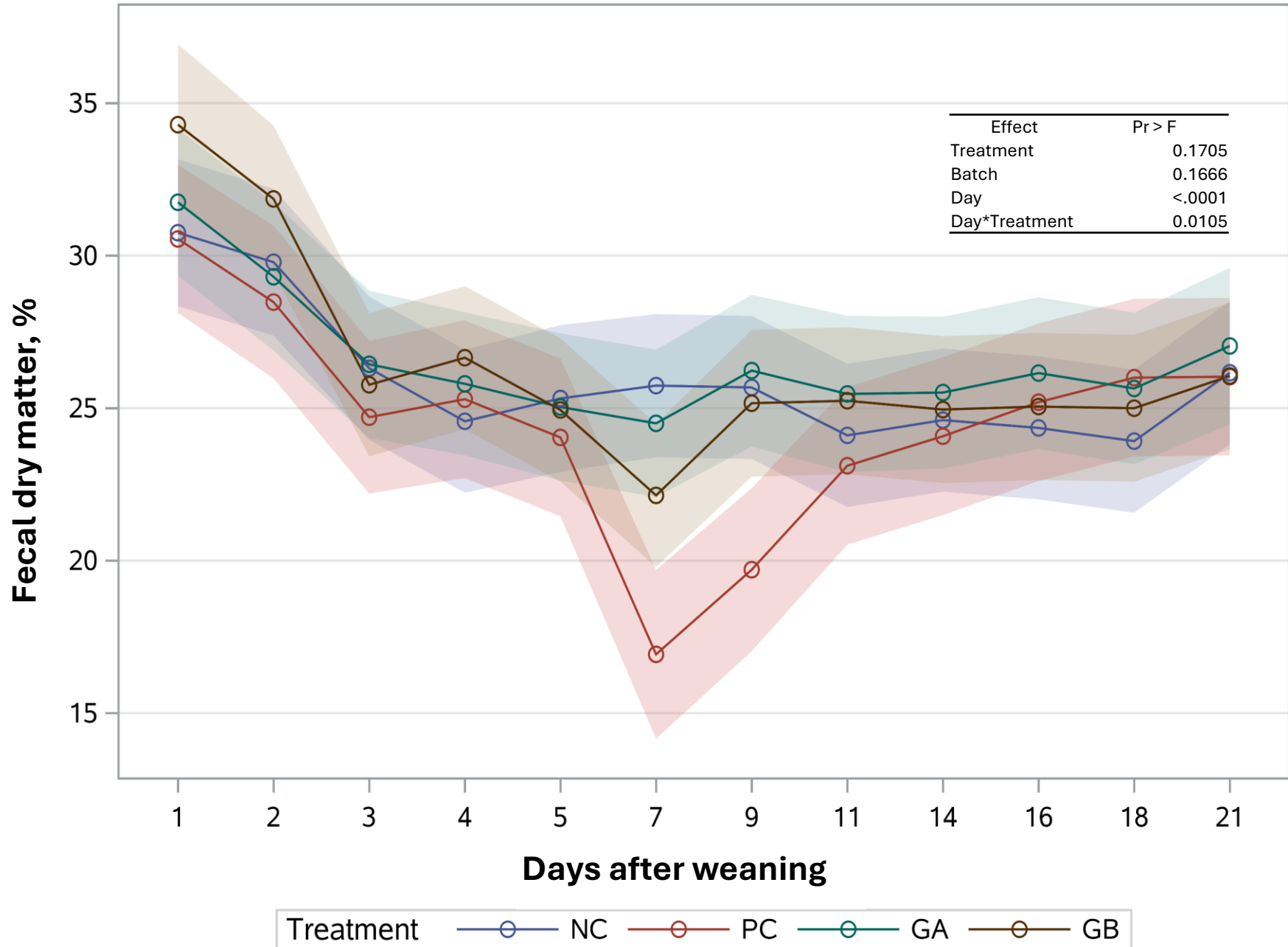

Supplement: Supplementary file 1 — Supplementary Material 1. [file 12866_2025_4247_MOESM1_ESM.pdf]

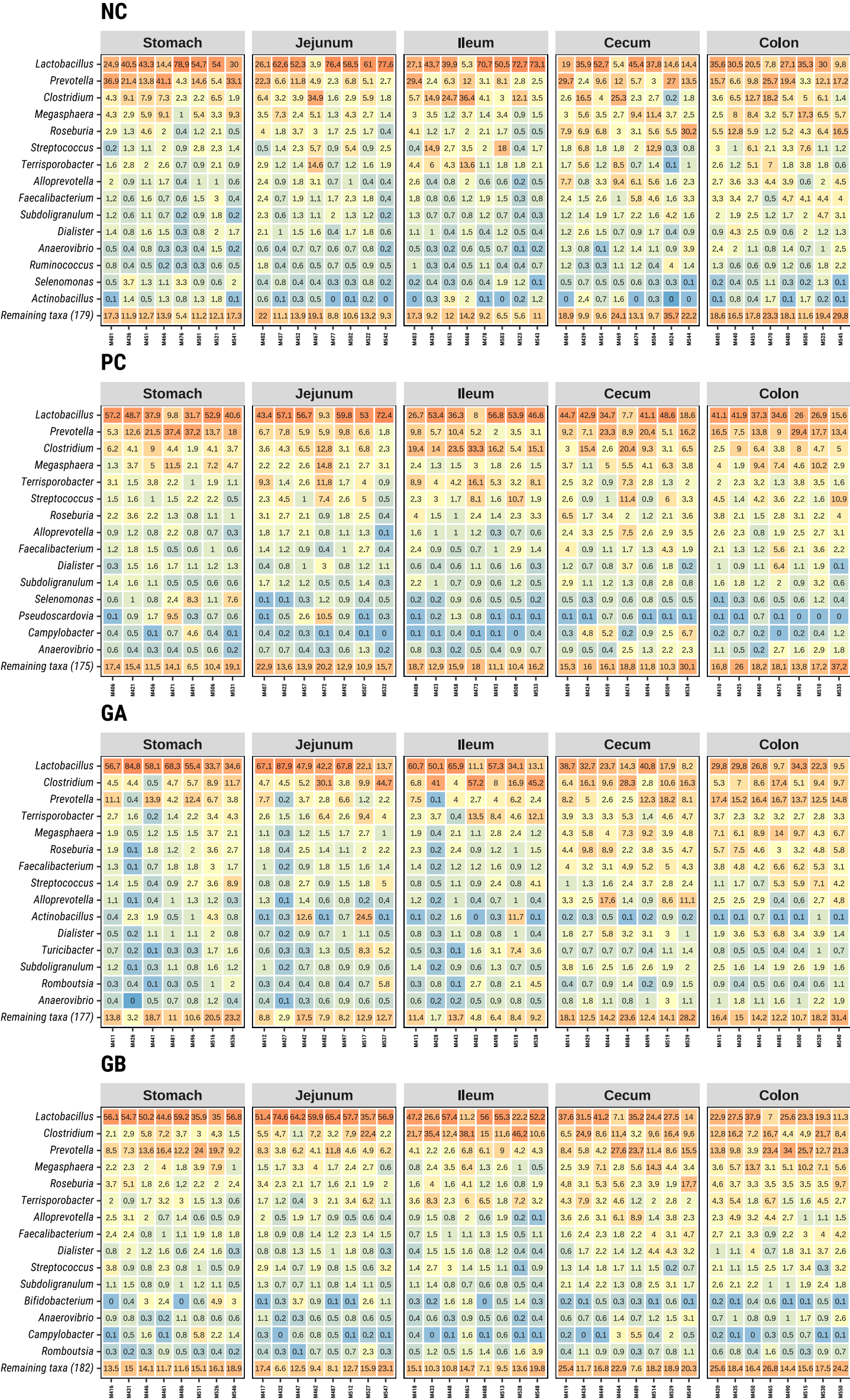

Supplement: Supplementary file 3 — Supplementary Material 3. [file 12866_2025_4247_MOESM3_ESM.pdf]
