# Supplementary material for: Effects of supplementation of garlic with apple pomace or blackcurrant on the gastrointestinal microbial ecosystem of organic pigs after weaning
Source: BMC Microbiol. 2025 Oct 2;25:608. doi: 10.1186/s12866-025-04247-2 (PMC12492707; doi:10.1186/s12866-025-04247-2)
Supplement: Supplementary file 2 — Supplementary Material 2. [file 12866_2025_4247_MOESM2_ESM.pdf]

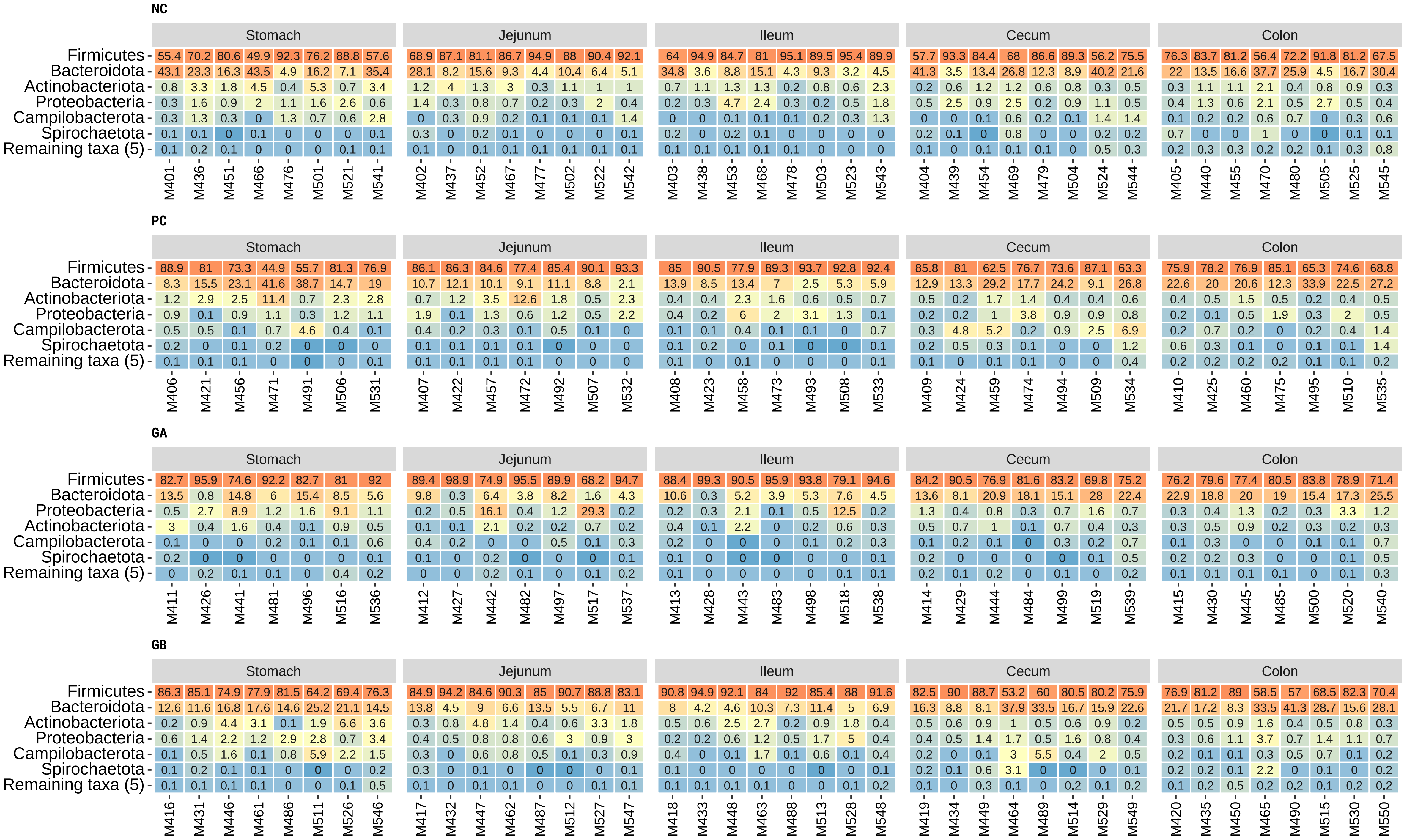

Figure S2 Relative abundance of bacterial phyla along segments of the gastrointestinal tract of individual pigs. The values indicate the relative abundance (%) of the six dominant phyla (Y-axis) across pigs (X-axis) and segments (Panels) in each treatment. NC: non-challenged and organic diet (n=8); PC: challenged and organic diet (n=7); GA: challenged, organic diet and garlic + apple pomace (3%+3%; n=7); GB: challenged, organic diet and garlic + blackcurrant (3%+3%; n=8). Mean values are presented in Figure 1 in main text.
