# Supplementary material for: Effects of supplementation of garlic with apple pomace or blackcurrant on the gastrointestinal microbial ecosystem of organic pigs after weaning
Source: BMC Microbiol. 2025 Oct 2;25:608. doi: 10.1186/s12866-025-04247-2 (PMC12492707; doi:10.1186/s12866-025-04247-2)
Supplement: Supplementary file 4 — Supplementary Material 4. [file 12866_2025_4247_MOESM4_ESM.docx]

**Table S1** Ingredient composition of the experimental diets (as-fed basis, %)

| Item, % | Diets^1^ | | |  |
| --- | --- | --- | --- | --- |
|  | NC - PC | GA | GB | |
| Organic wheat | 25.75 | 25.75 | 25.75 | |
| Barley | 22.25 | 22.25 | 22.25 | |
| Organic oats | 18.60 | 12.60 | 12.60 | |
| Garlic powder | 0 | 3.00 | 3.00 | |
| Apple Pulp powder | 0 | 3.00 | 0 | |
| Blackcurrant powder | 0 | 0 | 3.00 | |
| Fishmeal | 6.00 | 6.00 | 6.00 | |
| Organic soy cake | 5.70 | 5.70 | 5.70 | |
| Horse beans | 5.00 | 5.00 | 5.00 | |
| Organic rye | 5.00 | 5.00 | 5.00 | |
| Potato protein | 4.40 | 4.40 | 4.40 | |
| Organic barley | 2.80 | 2.80 | 2.80 | |
| Organic wheat bran | 2.00 | 2.00 | 2.00 | |
| Calcium carbonate | 1.15 | 1.15 | 1.15 | |
| Monocalcium phosphate | 0.47 | 0.47 | 0.47 | |
| Vitamin + Mineral premix^2^ | 0.40 | 0.40 | 0.40 | |
| Vitamin E | 0.2 | 0.2 | 0.2 | |
| NaCl | 0.28 | 0.28 | 0.28 | |

^1^ **NC**: nonchallenged and organic diet (n=16); **PC**: challenged and organic diet (n=13); **GA**: challenged, organic diet and garlic + apple pomace (3%+3%; n=14); **GB**: challenged, organic diet and garlic + blackcurrant (3%+3%; n=16).

^2^ Provided per kg of diet: 173 mg Fe (iron sulfate), 80 mg Cu (copper sulfate), 80 mg Cu (copper sulfate), 46 mg Mn (manganese oxide), 100 mg Zn (Zinc oxide), 0.30 mg I (calcium iodate), 0.30 mg Se (sodium selenite), 5400 UI vitamin A, 1000 IU vitamin D3, 215 IU vitamin E.
