# Supplementary material for: Effects of supplementation of garlic with apple pomace or blackcurrant on the gastrointestinal microbial ecosystem of organic pigs after weaning
Source: BMC Microbiol. 2025 Oct 2;25:608. doi: 10.1186/s12866-025-04247-2 (PMC12492707; doi:10.1186/s12866-025-04247-2)
Supplement: Supplementary file 5 — Supplementary Material 5. [file 12866_2025_4247_MOESM5_ESM.docx]

**Table S2** Ingredient composition of the experimental diets (as-fed basis, %)

| Item | Diets^1^ | | |
| --- | --- | --- | --- |
|  | NC – PC | GA | GB |
| Moisture, % | 10.64 | 10.12 | 9.57 |
| Crude protein, % | 21.40 | 21.77 | 21.21 |
| Gross energy, kcal/kg | 4476 | 4456 | 4451 |
| Crude fat, % | 4.06 | 4.15 | 3.79 |
| Ash, % | 4.96 | 5.07 | 5.20 |
| Indispensable amino acids, % |  |  |  |
| Arginine | 1.25 | 1.23 | 1.21 |
| Histidine | 0.48 | 0.48 | 0.46 |
| Isoleucine | 0.92 | 0.92 | 0.88 |
| Leucine | 1.59 | 1.57 | 1.52 |
| Lysine | 1.36 | 1.35 | 1.33 |
| Methionine | 0.40 | 0.38 | 0.38 |
| Phenylalanine | 1.02 | 1.00 | 0.97 |
| Threonine | 0.83 | 0.83 | 0.80 |
| Valine | 1.13 | 1.12 | 1.08 |
| Dispensable amino acids, % |  |  |  |
| Alanine | 0.98 | 0.97 | 0.94 |
| Aspartic Acid | 1.86 | 1.87 | 1.78 |
| Cysteine | 0.36 | 0.34 | 0.34 |
| Glutamic acid | 3.70 | 3.64 | 3.59 |
| Glycine | 1.00 | 0.98 | 0.96 |
| Proline | 1.29 | 1.28 | 1.25 |
| Serine | 1.02 | 1.00 | 0.98 |
| Carbohydrates, % |  |  |  |
| Fructose | 0.11 | 0.81 | 0.78 |
| Glucose | 0.25 | 0.77 | 0.53 |
| Sucrose | 2.74 | 3.12 | 3.02 |
| Fructans | 0.04 | 1.61 | 1.45 |
| Starch | 43.44 | 44.23 | 44.16 |
| *S-NSP^2^* | 3.57 | 2.35 | 3.60 |
| Rhamnose | 0.02 | 0.03 | 0.04 |
| Fucose | 0.01 | 0.01 | 0.02 |
| Arabinose | 0.59 | 0.51 | 0.67 |
| Xylose | 0.90 | 0.16 | 0.51 |
| Mannose | 0.20 | 0.18 | 0.22 |
| Galactose | 0.29 | 0.31 | 0.35 |
| Glucose | 1.27 | 0.65 | 1.36 |
| Uronic acids | 0.28 | 0.49 | 0.43 |
| *I-NSP^3^* | 11.73 | 11.88 | 12.13 |
| Rhamnose | 0.02 | 0.02 | 0.02 |
| Fucose | 0.02 | 0.02 | 0.03 |
| Arabinose | 1.73 | 1.63 | 1.61 |
| Xylose | 3.91 | 3.95 | 3.86 |
| Mannose | 0.26 | 0.38 | 0.30 |
| Galactose | 0.32 | 0.33 | 0.37 |
| Glucose | 1.23 | 1.83 | 1.31 |
| Uronic acids | 0.46 | 0.49 | 0.47 |
| Cellulose | 3.77 | 3.25 | 4.16 |
| Total NSP^4^ | 15.29 | 14.23 | 15.73 |
| Klason lignin | 3.00 | 3.41 | 2.79 |
| Dietary fiber^5^ | 18.33 | 19.25 | 19.96 |

^1^ Source of the values is Jerez-Bogota K, et al (2023) who analyzed and fed (in their study) the same fed in the current study. NC: non-challenge, standard diet; PC: challenged, standard diet; GA: challenged, Garlic and Apple pomace supplementation (3%+3%); GB: challenged, garlic and blackcurrant supplementation (3%+3%).

^2^ Soluble Non-Starch Polysaccharides

^3^ Insoluble Non-Starch Polysaccharides

^4^ Total Non-Starch Polysaccharides (S-NSP + I-NSP)

^5^ Total Non-Starch Polysaccharides + Lignin + Fructans
