# Supplementary material for: Effects of supplementation of garlic with apple pomace or blackcurrant on the gastrointestinal microbial ecosystem of organic pigs after weaning
Source: BMC Microbiol. 2025 Oct 2;25:608. doi: 10.1186/s12866-025-04247-2 (PMC12492707; doi:10.1186/s12866-025-04247-2)
Supplement: Supplementary file 6 — Supplementary Material 6. [file 12866_2025_4247_MOESM6_ESM.docx]

**Table S3** Growth performance of pigs from day 1 to day 21 (3 weeks postweaning).

| Treatment^1^ | Batch | | Body Weight, kg | | Average daily weight gain, g | Average daily feed intake, g | Gain: Feed |
| --- | --- | --- | --- | --- | --- | --- | --- |
|  |  |  | Day 1 | Day 21 |  |  |  |
| NC | 1 | | 20.17 | 34.20 | 664.99 | 1,330.74 | 0.50 |
| PC | 1 | | 19.99 | 33.97 | 654.21 | 1,364.73 | 0.50 |
| GA | 1 | | 20.61 | 32.75 | 596.19 | 1,199.41 | 0.52 |
| GB | 1 | | 19.81 | 32.30 | 574.37 | 1,170.35 | 0.49 |
| NC | 2 | | 20.61 | 35.39 | 721.52 | 1,345.99 | 0.53 |
| PC | 2 | | 20.82 | 35.34 | 719.55 | 1,237.39 | 0.53 |
| GA | 2 | | 20.24 | 34.16 | 663.04 | 1,221.38 | 0.54 |
| GB | 2 | | 19.86 | 33.82 | 647.17 | 1,247.25 | 0.52 |
|  |  |  |  |  |  |  |  |
| SEM^2^ | | | 1.326 | 1.098 | 52.295 | 55.263 | 0.018 |
| *P-*value^3^ | | |  |  |  |  |  |
| Treatment | | | 0.961 | 0.312 | 0.311 | 0.084 | 0.175 |
| Batch | | | 0.802 | 0.093 | 0.089 | 0.935 | 0.229 |
| Trt×Batch | | | 0.975 | 0.998 | 0.988 | 0.398 | 0.9817 |

^1^ **NC**: nonchallenged and organic diet (n=16); **PC**: challenged and organic diet (n=13); **GA**: challenged, organic diet and garlic + apple pomace (3%+3%; n=14); **GB**: challenged, organic diet and garlic + blackcurrant (3%+3%; n=16).

^2^ Pooled standard error of least squared means.

^3^ The models for body weight at day 21, average daily weight gain and average daily feed intake included the body weight at day 1 as model covariate (*P* < 0.001).
